# “It is an Issue of not Knowing Where to Go”: Service Providers’ Perspectives on Challenges in Accessing Social Support and Services by Immigrant Mothers of Children with Disabilities

**DOI:** 10.1007/s10903-014-0122-8

**Published:** 2014-11-07

**Authors:** Nazilla Khanlou, Nasim Haque, Sinead Sheehan, Gail Jones

**Affiliations:** 1 Faculty of Health, York University, Toronto, ON Canada; 2 Kerry’s Place Autism Services, Mississauga, ON Canada

**Keywords:** Immigrant, Social support, Service providers, Disability, Mothers

## Abstract

In Canada little is known about the challenges immigrant mothers of children with disabilities encounter in accessing formal and informal social support. This paper presents the perspectives of service providers on the mothers’ challenges. Data was collected from 27 service providers in Toronto, Canada in 2012 through in-depth interviews. The interview guide was informed by published literature on families of children with special needs. Level one analyses entailed descriptive analyses; and level two consisted of applying House’s 4 domains of social support to organize the themes. Following House’s domains, challenges to (1) Structural support, (2) Instrumental support, (3) Emotional support, and (4) Perception of support were identified. Among providers who work with families of children with disabilities there is recognition of the mothers’ particular challenges in light of their immigration status. Language and communication are significant barriers for immigrant mothers in accessing social support.

## Introduction

Immigration is important to Canada’s population growth with approximately 250,000 immigrants arriving annually. The province of Ontario is home to about 40 % of all permanent migrants [1]. In 2011, immigrants living in Toronto accounted for 49 % of the population, one-third being newcomers having arrived in Canada in the last 10 years [2]. There is also growing ethno-cultural diversity. For example, 49 % of Toronto’s residents identified themselves as a visible minority [2] and about 2.24 % of Ontario’s 12 million residents reported not being able to speak English or French [3]. Research studies indicate that ethno-cultural factors influence access to health care [4, 5].

Mothers of children with disabilities rely on and access multiple services simultaneously for the growth and development of their child. For example, they may concurrently access services involving schools, child care therapies, medical and other social services such as respite care. Access to care is highlighted in the literature as one of the limiting factors for minority groups unable to receive adequate services [6]. As the population becomes more ethno-culturally diverse, service delivery systems are increasingly being challenged to provide culturally appropriate services to diverse groups of non-English and non-French speaking clients. Previous studies have examined the differences in utilization and access to health and social services between Canadian born, immigrants, and refugees. However, few studies have examined service providers’ perspective on the challenges encountered by immigrant mothers of children with disabilities in accessing social support.

Across Canada, 202,350 (3.7 %) children under the age of 15 years and 102,680 (2.5 %) of all children between the ages of 15 and 24 years have one or more disabilities [7]. The number of children with disabilities growing-up within immigrant families in Canada is not well documented. However, studies from United Kingdom and Australia indicate that immigration may be a factor in the rising rates of autism in Europe and other countries. For example, Keen, Reid & Armone (2010) [8] reported that immigrant mothers who were born outside Europe had a significantly higher risk of having a child with an autism-spectrum disorder as compared with mothers born in the UK. They noted that the highest risk observed was for the Caribbean group. Raising a child with a disability has multiple consequences in all spheres of family life [9–11] including social implications [12]. The challenges faced by the families are complex and we currently do not have a sufficient understanding of the specific nature and extent of disability related health and social support needs [13] of the families. While taking care of children with disabilities has multiple challenges for all mothers, being an immigrant mother intensifies these challenges. As a result, gaps in access to essential social support remain rampant, even when services exist.

To help bridge the gap we conducted the Mothers Project (Social Support for Immigrant Mothers of Children with Disabilities). The objectives of the Mothers Project were to understand the social support experiences and service needs of immigrant mothers of children with disabilities, and to investigate service providers’ perspectives on the challenges faced by immigrant mothers in accessing social support and services. We present in this paper findings on the perspectives of service providers.

## Methods

### Study Design

The qualitative descriptive study was carried out in the Greater Toronto Area (GTA) and data was collected between April and December, 2012. A single stage purposive sampling strategy [14] was used to select participants from diverse service organizations in the GTA. Ethics approval for the study was obtained from York University’s Research Ethics Board, Toronto, Ontario. For consistency and to add rigor to the study, all interviews were conducted by the same interviewer (who was also the project coordinator).

Invitation correspondence was sent to selected service providers. Participants who responded and agreed to participate were subsequently contacted by the project coordinator to further explain the project and to seek their permission to send the consent form for their review. At the beginning of each interview, the interviewer obtained verbal consent from each participant. All interviews were conducted in English using an open-ended interview guide.

### Interview Guide

The interview guide was designed to explore providers’ perspectives on the challenges immigrant mothers encountered in accessing social support and services (Appendix 1). It was informed by published literature focussed on working with families of children with special needs [15, 16] and by the research team’s own experience in working with immigrant populations and families of children with disabilities in Canada.

### Participants

In total, 27 service providers volunteered to participate in the study. Participants were recruited from service organizations representing social work, health services, legal services, community services. The inclusion criterion was that the service provider was engaged in providing services to families of children with disabilities. Participants volunteered their time and were not compensated. They were later sent a thank you correspondence by the principal investigator (PI) as an appreciation for their time.

### Data Collection and Analyses

Telephone interviews were conducted using open ended questions asking for details of services provided, provider perceptions of the difficulties mothers encountered, and challenges the providers faced in providing services to the mothers (Appendix 1). Telephone interviews were completed in 60–90 min, and transcribed by the interviewer using Dragon Naturally Speaking software. Following each interview, field notes were taken by the interviewer regarding her impressions about the interview. These notes were later discussed with the PI and with other researchers. This process helped in contextualizing the findings through additional insight beyond that provided by the text of the transcripts. For example, by creating connections between what the interviewee said to existing policies and practices, or to similarities in challenges in social support observed in other interviews, or to the interviewer’s impressions regarding how effectively an interview took place.

Two levels of analyses took place. During the first level, as data was still being collected, the PI and two researchers from the research team independently reviewed the transcripts of the completed interviews and generated preliminary themes and sub-themes. The three then met face-to-face along with the research staff to discuss similarities and differences. This process of coding separately by the three researchers and then meeting to discuss findings took place two more times. The resultant first level analyses were descriptive and followed Sandelowski’s (2000, 2010) qualitative description approach [17, 18]. During the first level of analyses, the three researchers’ positionality no doubt influenced the emerging themes and subthemes. Collectively, the three came from 2 disciplines (nursing and sociology), 2 were from migrant backgrounds, and each had different levels of experience in immigration and disabilities studies research. All three researchers had prior experience in qualitative research.

During the second level, the themes from the first level were linked to House’s 4 domains of social support [19], which we have found a useful format of presenting research findings to diverse audiences, including policy makers, practitioners, immigrant parents, and scholars. As per House, we recognized social support as a multidimensional construct that included: (1) *Structural support* (availability of and access to services or information about disabilities); (2) *Instrumental support* (tangible support, such as financial support or care-giving and respite services); (3) *Emotional (expressive) support* (may be offered by friends, family and compassionate professionals); and (4) *Perceptive support* (individual’s view on whether the support provided is sufficient and helpful).

## Results

Of the 27 service providers, 22 (81 %) participants were female and 5 (19 %) were male participants. The mean years of work experience was 14 years (range 2–35 years) in their respective professions (Table 1). Participants worked as social workers, family physicians/paediatricians, counsellors, program managers and lawyers. They were employed in various organizations such as community centers, social work, children treatment centers, and legal services (Table 1).

**Table 1:** Characteristics of service providers

Characteristics	Service providers (n = 27)
Gender of participants	
Male	5 (19 %)
Female	22 (81 %)
Years of experience	
Mean (years)	14
Range (years)	2–35
Occupation	
Family physician/paediatrician	2 (7 %)
Social workers	8 (30 %)
Family counsellors/mediators	3 (11 %)
Manager/supervisor (service centers)	6 (22 %)
Child protection immigration specialist	1 (4 %)
School management	1 (4 %)
Policy makers	1 (4 %)
Lawyers serving persons with disabilities	5 (19 %)
Types of organizations	
Children treatment/rehabilitation centers	4 (15 %)
Community health centers	2 (7 %)
Paediatric units of teaching institution	2 (7 %)
Ombudsman/child advocacy/legal aid office	8 (30 %)
School related	2 (7 %)
Parents support group	2 (7 %)
Multiservice centers	3 (11 %)
Autism/epilepsy/family physician clinics	3 (11 %)
Provincial parliament	1 (4 %)

### Structural Support

Availability of services (structural support) in and of itself does not mean there is equal access to it. Participants discussed the many barriers to services and lack of information faced by immigrant mothers. Often language and communication barriers impacted access to and effectiveness of other forms of support.

#### Language and Communication Barriers

Service providers identified English as a second language and communication as a key challenge for immigrant mothers in accessing services. One service provider (SP) said: “The language issue is huge if the person is not comfortable with English” (SP 13). Providers stated that many mothers had difficulty in communicating with SPs and understanding the health care system, for example, the importance of accessing simultaneously different SPs. They also reported that some mothers did not understand the medical terminologies used by providers, for example, many mothers did not understand the meaning of respite care and therefore were not able to ask for the services they needed.

#### Navigating New Systems

Service providers discussed Canada’s complicated service systems and the difficulty immigrant mothers had in understanding and navigating the new system. One provider stated: “The system is really complicated. Even for me, it is really complicated as a professional” (SP 7). Another provider explained:

There is a general lack of understanding of our system. Families were unsure where they can go to access resources and to investigate services. It is very difficult for them to get such information (SP 3).

#### Excessive Paper Work

Providers emphasized that for availing the needed services, organizations required mothers to complete long and complicated forms in English. For many mothers, the lack of professional help, time constraints, and limited language skills were barriers to completing the needed paperwork to access services. Providers suggested that the system should provide someone to work with these mothers to help them in completing the required forms:

The main difficulty is that forms are very long and complicated… It is discouraging. It is like a full-time job, there is so much work to coordinate…..What the mothers need is someone who will sit down with them and fill out forms for them and provide them with advice (SP 7).

#### Dispersed Services

Providers reported that the services needed for the optimal growth and development of the child were dispersed and located in different parts of the city, making it difficult for new immigrant mothers to access them. Mothers usually did not have access to personal transportation and required to use public transit. In addition, communication barriers made it difficult for mothers to recognize the need for several providers’/service agencies’ simultaneous involvement in providing care to their children.

The name of service providers is very confusing. There are many different agencies. There is an overlap and there are gaps in service provision…. It is an issue of not knowing where to go. We need one central place of access (SP 7).

Participants suggested that appointing a case worker for each family could resolve many of these issues. One provider described:

Many of my clients do not have a caseworker or social worker to work with. What is needed is a central person to whom immigrant families can go to ask for what is available (SP 3).

#### Cultural Differences

The cultural values and beliefs of the mothers’ home country may also affect access of services. Participants noted that immigrant mothers do not ask for services but are instead grateful of the service that is provided to them. Most immigrant mothers do not realize that access to health and education are their basic rights and, therefore, do not forcefully seek the resources they need for their children. One SP described her experience:

There are cultural issues. People [immigrant mothers of children with disabilities] must be aggressive and assert themselves. Many feel uncomfortable [because they think the services they receive is a privilege]. Things can get way out of hand and then they need support. Immigrant parents are not sure what their rights are and so it’s easier to push them aside (SP 26).

Mothers’ cultural beliefs and values may also influence how they follow up with treatment and professional supports, and how they view their child’s disability. In some cultures, disability is associated with stigma and a sense of shame:

Disability is a social taboo in some cultures…. Some individuals will blame the child for being disabled. Some communities blame the women for having had a disabled child (SP 11).

If this stigma is not adequately addressed, some mothers may face social isolation and social exclusion.

#### Limitation of Services

Participants also spoke about skill limitations. They reported that some SPs had inadequate information of the needs of immigrant mothers of children with disabilities, especially for children with severe disabilities. They also reported that some providers were also not aware of all the services and resources that are available for families of children with disabilities. A provider explained:

Disability awareness as a whole is a problem. Yes, there is a special needs unit. [But] some children aid’s society workers don’t know what assistance is available for children with severe disabilities! They need to know more about that whole world (SP 18).

### Instrumental Support

House describes instrumental support as tangible aid and services provided by social institutions including financial assistance, material goods or services [19]. Instrumental support can also be provided through support from family, friends, extended family, informal networks and service providers through care-giving and respite.

#### Social Support from Family and Friends

Social support is a major adaptive resource for mothers of children with disabilities. It acts as a protective factor and helps mothers to ease their stress and adjust to life with their child [15, 20]. As most childhood disabilities by nature involve lifelong disability and support needs, the presence of informal supports through family and friends can often make a powerful impact on the quality of life for the child and family involved. SPs will come and go and change jobs, but family and friends can create consistency and predictability in care over the years. When immigrants enter their new country of residence, they lose the family and social ties they had in their country of origin putting mothers at a higher risk of isolation and greater distress. Data from Statistics Canada also underlines the importance of the help parents received from informal sources, such as extended family, friends, and neighbours [7]. Providers described the difficulties faced by immigrant mothers who did not have extended families or friends. One provider described:

It is devastatingly difficult to care for these children. Immigrant mothers are less likely to carpool, less likely to have friends who could pick up groceries for them [and over time because of intense demands] friends fade away (SP 20).

### Emotional Support

Emotional support is the warmth and attention provided by sources of social support. It involves the offering of empathy, concern, affection, trust, acceptance, encouragement or caring when needed [21]. Immigration can cause changes within family relationships given the downward employment of newcomer families. While fathers seek employment or are underemployed (therefore working more than one job), mothers can be the sole parent who provides care-giving to their children with disabilities. Some immigrant mothers may not have the support they need from their partners, making it difficult for them to make decisions when they seek care for their children. For example, one provider explained:

In……[some] culture, the father has special place. Mother is a 24/7 caregiver, but the father is making decisions. This style is challenging sometimes because the father may not be realistic in his expectations for the child’s behavior, and challenges for the child remain unseen (SP 2).

### Perception of Support

The fourth dimension of House’s conception of social support refers to how helpful the support offered is perceived to be by its recipient. SPs interviews pointed both toward the mothers’ resilience as well as their support needs. The extra-ordinary parenting needed in the context of raising children with disabilities was recognized:

They have a lot of strengths. Care and concern for their child with a disability regardless of what they are going through… I really marvel at them… You can see them blossoming with their relationship building. And with ESL they get more confident. [This] helps them with advocacy, then they get the information themselves (SP 16).

However, immigration brought with it language and cultural barriers that influenced the support received and perceived:

The families who have children with disabilities are using all their energy in caring for their children and to get through the system and so to be doing this with language or cultural barriers would be so difficult - it would be so much (SP 11).

## Discussion

This study examined service providers’ views on the challenges immigrant mothers of children with disabilities encounter in regards to social support. The results highlight that among SPs who work with families of children with disabilities there is recognition of the particular challenges faced by these families in light of their immigration status. The findings bear relevance to practice, policy, and research literature. Consistent with past research on ethnic minority groups who encounter language barriers when accessing social support and health services, our findings indicate that language and communication are significant access barriers for immigrant mothers. Communication between clients and SPs is fundamental to social services and health-care access and delivery [22]. Many recent immigrant mothers speak and understand English as a second language, which creates difficulty in interacting with and understanding professionals. This lack of understanding from both parent and provider can result in misinterpretations of diagnosis, difficulties in accessing necessary services, and challenges in receiving appropriate treatment [22–27]. Moreover, medical language is often complex and difficult to understand, which adds further stress for these parents who may not completely understand the specifics of their child’s illness [23].

Upon initial years of resettlement, mothers have difficulty in understanding and navigating complex systems of service provision. Extensive paper work and accessing geographically dispersed services are hurdles mothers face on an ongoing basis. To address these issues, some of the providers argued for a case worker model, a person who works with the mothers to help them understand the systems of service, complete paper work for them, and inform them about available resources. Case management services exist in Canada for community-based mental health care [28] and home-based nursing care [29]. In 2012, the Canadian government announced funding for a National Case Management Network whose goal is to develop standardized knowledge and skills for case management [30].

Participants noted how different cultures perceived disabilities and its effect on mothers accessing the services. Previous studies have recommended a fuller appreciation of the cultural diversity [22, 24] in our society and suggested to have more ethnically and linguistically diverse staff who are well-trained in cultural competence. They further suggest that in bigger cities, where most of the immigrant population settles, an attempt should be made to match service providers with clients of similar cultural backgrounds.

SPs also spoke of their own professional informational needs and argued that some providers were not well informed about the services and resources available for children with disabilities. Indeed all providers should have increased awareness and knowledge of the needs of their clients through continuous education and training. This could be accomplished through regular continuing education programs. These training programs should include training in the area of sensitivity to cultural diversity.

The association between instrumental and emotional social support and health is well recognized in the literature as an important social determinant of health through which individuals attain emotional and practical resources that can support their wellbeing [31, 32]. SPs in our study alluded to immigrant mothers’ experiences of lack of social support from their partners, families and friends after migration mostly because new immigrants leave behind extended social networks in their country of origin when they migrate. Others who may have the networks can feel a strong stigma about the disability of their child by their own support systems, who tend to blame the parents for the child’s condition [33, 34]. Lack of social support from family and friends can increase the burden of care, reduce social interaction and push mothers to isolation, and thus reduce the likelihood of accessing social support and services for their child [33, 35].

In many cultures gender roles and the division of household labour is well defined. Mothers view their parental caregiver role as primary, and therefore experience great parental strain through caretaking activities for their child with disability. They take the sole responsibility of handling specific medical and social aspects of their child’s disability, and spend most of their time with the child, which adds to their stress and sadness [36]. After immigration mothers expect their partner would aid with household chores and with the medical care of the child but when these expectations are not fulfilled it further adds to their stress and frustration [37].

### Limitations of the Study

The findings reported from this qualitative study are based on purposive sampling of service providers who volunteered to participate in the study. Therefore, the extent to which the participants’ views are representative of all SPs serving immigrant mothers of children with disabilities is unknown. However, given the number of participants and range of professions represented, findings can point the way for future quantitative studies focussing on service providers’ perspectives and practice patterns. In addition, this study was carried out in the GTA and it is likely that service systems in other big cities can operate differently, indicating the need for cross-city and possibly cross-province comparisons. However, through national and local presentations of our findings to providers and mothers on several occasions, we have received support of our findings. In addition, as part of member-checking we sent the draft of the study’s report to SP participants for their input.

### New Contributions to Literature

This article presents perspectives of service providers. While stressors to mothers’ mental wellbeing have been examined across studies and service barriers documented from mothers’ perspectives, scant attention has been given to what service providers identify as barriers to social support for immigrant mothers in the context of caregiving and parenting their children with disabilities.

The findings of this study suggest that language and communication are significant barriers for immigrant mothers in accessing social support and services. The issue persists even when interpretation services are available. Future epidemiologically designed studies are needed to examine the characteristics of the families so that local programs can address their specific translation and cultural interpretation needs. Focusing on removing language and communication barriers in developing culturally responsive service delivery mechanisms for non-English/French speaking immigrant mothers of children with disabilities is needed. The study’s findings also indicate that service providers recognize barriers to structural support as among the prevalent challenges immigrant mothers of children with disabilities encounter in accessing social support.

## Appendix 1: Service Providers’ Interview Guide

### Demographic Questions

1. What is your occupation?
2. What kind of organization employs you?
3. How long have you been doing this kind of work?

### Open Ended Questions for Service Providers Across Sectors

1. In what context do you have contact with immigrant mothers of children with disabilities? (School board, hospital, etc.).
2. In your experience, what would be the most significant barrier for immigrant mothers in accessing services and supports for their children with disabilities?
3. In the sector in which you work/practice, what do you perceive would be of most help to these mothers in meeting the needs of their children?
4. Can you tell me about what supports are already in place for immigrant mothers of children with disabilities?
5. On the whole, how do these mothers seem to you? (oppressed/coping).
6. In your sector are these mothers more or less or equally involved with children’s aid societies and to what extent do you understand their involvement to be linked to their being the mother of a child with a disability?
7. Are you aware of any support groups offered through your work place for immigrant mothers of children with disabilities?
8. In the sector in which you work/practice, what do you perceive would be a help to these mothers in meeting their own needs as mothers with children with additional or complex care needs?
9. In what areas do you see immigrant mothers of children demonstrating strengths in terms of accessing what their children require?
10. Do these mothers address complications with professionals in your field (at your school/hospital/social service occupation etc.) directly and if so how?
11. Are there any groups of immigrant mothers who appear, in your experience, to be experiencing a higher degree of marginalization or who have more difficulties in accessing services and supports for their children with disabilities?
